# *In-silico* characterization and structure-based functional annotation of a hypothetical protein from *Campylobacter jejuni* involved in propionate catabolism

**DOI:** 10.5808/gi.21043

**Published:** 2021-12-31

**Authors:** Lincon Mazumder, Mehedi Hasan, Ahmed Abu Rus'd, Mohammad Ariful Islam

**Affiliations:** 1 Department of Microbiology, Jagannath University, Dhaka 1100, Bangladesh; 2 icddr,b, Mohakhali, Dhaka 1212, Bangladesh

**Keywords:** *Campylobacter jejuni*, functional annotation, homology modeling, hypothetical protein, *in-silico* characterization, propionate catabolism

## Abstract

*Campylobacter jejuni* is one of the most prevalent organisms associated with foodborne illness across the globe causing campylobacteriosis and gastritis. Many proteins of *C. jejuni* are still unidentified. The purpose of this study was to determine the structure and function of a non-annotated hypothetical protein (HP) from *C. jejuni*. A number of properties like physiochemical characteristics, 3D structure, and functional annotation of the HP (accession No. CAG2129885.1) were predicted using various bioinformatics tools followed by further validation and quality assessment. Moreover, the protein-protein interactions and active site were obtained from the STRING and CASTp server, respectively. The hypothesized protein possesses various characteristics including an acidic pH, thermal stability, water solubility, and cytoplasmic distribution. While alpha-helix and random coil structures are the most prominent structural components of this protein, most of it is formed of helices and coils. Along with expected quality, the 3D model has been found to be novel. This study has identified the potential role of the HP in 2-methylcitric acid cycle and propionate catabolism. Furthermore, protein-protein interactions revealed several significant functional partners. The *in-silico* characterization of this protein will assist to understand its molecular mechanism of action better. The methodology of this study would also serve as the basis for additional research into proteomic and genomic data for functional potential identification.

## Introduction

As a human diarrheal pathogen *Campylobacter jejuni*, a well-known gram-negative bacterium, was first identified in 1973 [1]. It has several features like thermophilic, microaerophilic, no fermenting, non-spore forming, motile, single flagellum properties [2]. *C. jejuni* is a common foodborne pathogen that causes acute gastroenteritis in people globally and is prevalent in developed countries [1,3]. The incidence of infection by *C. jejuni* is more frequent than the infections caused by other common species including *Escherichia coli* O157:H7, *Salmonella* and *Shigella* [4]. *C. jejuni* possesses remarkable distinctive biochemical features from other microbial species including alpha-hemolysis, catalase sensitivity, hippurate hydrolysis, and nitrate reduction [5].

The genome of *C. jejuni* is made up of 1,641,481 base pairs containing 1,707 genes which are predicted to encode 1,654 proteins [6]. The functions of several of these proteins are still unknown. Uncharacterized protein families and domains of unknown functions both include proteins with uncertain functions [7]. For these reasons, the research interest for several unknown proteins of *C. jejuni* has increased among biological researchers. These unknown proteins, originated from an open reading frame with no experimental evidence of translation, are termed as hypothetical proteins (HPs) due to lack of functional annotations [8].

Over the last few decades a revolution in computational biology has led to the development of numerous servers and tools to aid in the prediction of protein function. HPs that have unknown features can be identified by virtue of their homology to known proteins [7]. A number of bioinformatics tools including the CD Search Service, InterProScan have been designed to specify functions of HPs from many bacterial species [9]. Furthermore, the study of protein-protein interaction (PPI), which play an essential role during cellular processes, is crucial to understand the function of a protein in a biological network using software such as the STRING database [10]. Three-dimensional (3D) modeling, however, is also important to correlate structural knowledge with the function of undetermined proteins, through homology searches at the Protein Data Bank [11].

The aim of this study was to ascribe structural and biological function of the HP NVI_CJUN_00861 (accession No. CAG2129885.1) of *C. jejuni*, involved in catabolism of a short chain fatty acid (SCFA). Among SCFAs found within the gut, *C. jejuni* metabolizes only acetate and lactate [12]. Therefore, a protein involved in metabolism of a SCFA will provide insight about the metabolic flexibility of *C. jejuni*. A number of *in-silico* techniques were used to predict the physicochemical properties, phylogenetic information, subcellular distribution, secondary and 3D structure, active site location, functional properties, and PPI of the HP engaged in metabolism.

## Methods

### Sequence retrieval and phylogeny analysis

The amino acid sequence of the HP (accession No. CAG2129885) from the bacteria *Campylobacter jejuni* was retrieved as FASTA format from the NCBI protein database (https://www.ncbi.nlm.nih.gov). We have reviewed all bioinformatics tools and databases used in this study for functional annotation of HP (Table 1). To analyze the sequence similarity, BlastP [13] was used. The MUSCLE v3.6 [14] was used to conduct multiple sequence alignment and MEGA X [15] to phylogenetic analysis.

### Physicochemical properties analysis

The ProtParam (http://web.expasy.org/protparam) [16] tool of the ExPASy server was used to analyze the physicochemical properties of the protein. The ProtParam tool computes various physicochemical properties such as molecular weight, theoretical isoelectric point (pI), composition of amino acid, total number of positive and negative residues, instability index, aliphatic index (AI), grand average of hydropathicity (GRAVY), molecular formula, and estimated half-life.

### Subcellular localization identification

The subcellular localization was anticipated by utilizing the CELLO server (http://cello.life.nctu.edu.tw) [17]. The results were further cross-checked by using PSLpred (http://crdd.osdd.net/raghava/pslpred) [18] and PSORTb (https://www.psort.org/psortb) [19] servers which are used to predict subcellular localization of bacterial proteins.

### Secondary structure prediction

The Self-Optimized Prediction Method with Alignment server- SOPMA (https://npsa-prabi.ibcp.fr/cgibin/npsa_automat.pl?page=npsa_sopma.html) [20] was used to predict the studied protein's secondary structure. The result was cross-checked by using PSI-blast based secondary structure predicting PSIPRED server (https://bioinf.cs.ucl.ac.uk/psipred) [21].

### 3D structure prediction and quality assessment

The 3D model of the protein was generated by the HHpred server (https://toolkit.tuebingen.mpg.de/tools/hhpred) [22]. The YASARA energy minimization server (http://www.yasara.org/minimizationserver.htm) [23] was utilized to increase the side-chain accuracy, physical realism, and stereochemistry in homology modeling. The PyMOL v2.0 [24] was used for structural analysis and model figure generation. The SAVES server (https://services.mbi.ucla.edu) was used to assess the HP's anticipated 3D structure model's reliability. The Ramachandran plot analysis [25] in PROCHECK was used to visualize the backbone dihedral angles ψ against φ of amino acid residues in the HP structure, Verify3D [26] to determine the compatibility of an atomic model (3D) with its amino acid sequence, and ERRAT [27] to cross-check the studied HP structure.

### Functional annotation

To identify the conserved domain of the protein sequence, the Conserved Domain Search Service (CD Search) (https://www.ncbi.nlm.nih.gov/Structure/cdd/wrpsb.cgi) [28] from NCBI was used. The protein sequence analysis and classification server InterProScan (https://www.ebi.ac.uk/interpro/search/sequence) [29] was then used for the functional analysis of the protein.

### Protein-protein interaction

PPIs are a never-ending, intricate web of reactions that are essential for the control and execution of most biological processes. A protein-protein functional interaction network was identified by the STRING v11.0 (https://string-db.org) [10] search.

### Active site identification

The active site of the HP was identified by the Computed Atlas of Surface Topography of Protein (CASTp) (http://sts.bioengr.uic.edu/castp) [30] which is an online asset for finding, outlining, and estimating inward surface regions on protein 3D structure.

### Performance assessment

A receiver operating characteristic (ROC) was carried out for randomly selected 40 proteins with known functions of *C. jejuni* (Supplementary Table 1) to confirm the accuracy of the predicted function of the HP using the same bioinformatics tools and databases that were used. We used two binary numerals “0” and “1” to classify the prediction as true positive (1) and true negative (0) whereas the integers (2, 3, 4, and 5) to evaluate the six levels diagnostic efficacy. The classification data were submitted to a web-based calculator to calculate the sensitivity, specificity, ROC area, and accuracy of the tools used to annotate the function of HP [31].

## Results and Discussion

### Sequence and similarity information

**Fig. 1.**
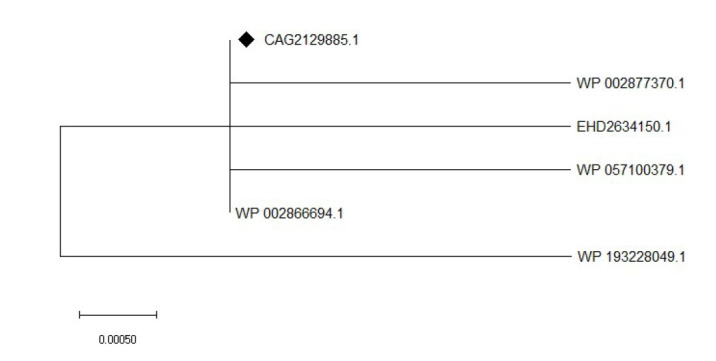
Phylogenetic relatedness of the study protein (indicated with a black diamond) along with similar other proteins obtained from non-redundant protein sequences (nr) database. Scale bars represents substitutions per nucleotide site. Evolutionary analyses were conducted in MEGA X using Jones-Taylor-Thornton model with 1,000 bootstraps.

All information of the HP (accession No. CAG2129885) was collected from the NCBI database (Supplementary Table 2). BlastP was performed against the non-redundant protein sequences (nr) database and UniProtKB/Swiss-Prot (swissprot) database which showed demonstrated homology of the HP with other MmgE/PrpD family protein and cis-aconitate decarboxylase (CAD), respectively (Tables 2 and 3). A phylogenetic tree (Fig. 1) was constructed using the neighbor-joining method with a bootstrap replication of 1,000 to confirm the homology assessment between proteins.

### Physicochemical features

The physicochemical properties of the studied protein (Supplementary Table 3) were obtained from the ExPASy ProtPram server illustrated that the protein contains 446 amino acids with a molecular weight of 49478.88 Da. Among the composition Ala (46), Ile (42), Leu (42), Lys (38), Phe (33), Ser (31), Asn (30), Glu (29), Gly (26), Asp (24), Val (17), Thr (14), Pro (14), His (13), Tyr (11), Gln (10), Cys (8), Met (8), Arg (7), and Trp (3) were most abundant. The number of negatively charged residues (Asp + Glu) and positively charged residues (Arg + Lys) was computed as 53 and 45, respectively. The pI was calculated as 5.93, which is an indicator that the protein is acidic (pH < 7) in character. The instability index was found to be 29.84 which classifies the HP as a stable protein. The AI was found to be 94.82 which implies the stability of the protein over a wide range of temperatures. The GRAVY score, ‒0.002, indicated that the protein is soluble in water (hydrophilic). The molecular formula of the HP was C_2250_H_3490_N_574_O_649_S_16_. The putative protein's half-life was estimated to be >20 h in yeast (*in-vivo*), >10 h in *Escherichia coli* (*in-vivo*), and 30 h in mammalian reticulocytes (*in-vitro*).

### Subcellular localization

Since protein subcellular localization can provide information about a protein's function in an organism, computerized prediction of protein subcellular localization is an important technique for protein analysis and annotation. Subcellular localization involves the identification of the protein location within a cell. The protein functions are greatly influenced by their subcellular localization. Based on analysis of the CELLO server protein localization predictions, the HP was identified as a cytoplasmic protein. The PSORTb server also identified the protein as a cytoplasmic one with a high localization score (9.97). The PSLpred protein subcellular localization server similarly indicated the protein as a cytoplasmic one.

### Secondary structure analysis

**Fig. 2.**
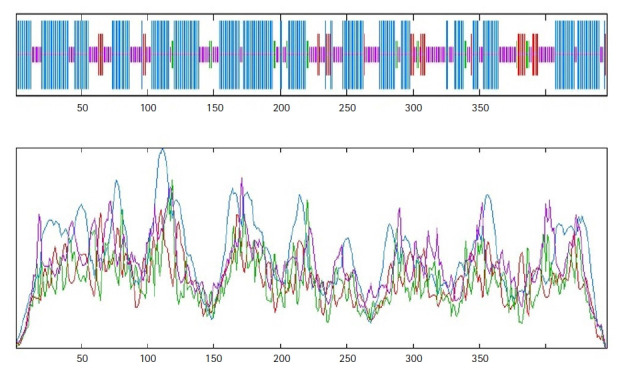
Secondary structure model predicted by the SOPMA server.

**Fig. 3.**
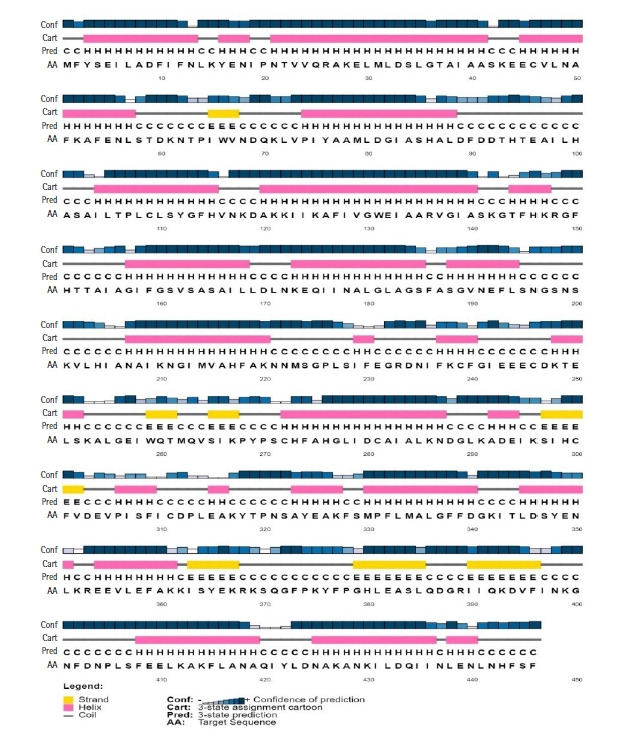
Secondary structure model by PSIPRED server.

Protein function is highly conserved by its structure. A significant portion of the secondary structure of the protein is helix, sheet, turn, and coil. The secondary structure of the HP, obtained from SOPMA server, demonstrated that it was composed of the alpha helix (55.16%), random coil (33.41%), extended strand (7.17%), and beta-turn (4.26%) (Fig. 2). A similar result was found from the PSIPRED server (Fig. 3) validated the previous result.

### 3D structure analysis

**Fig. 4.**
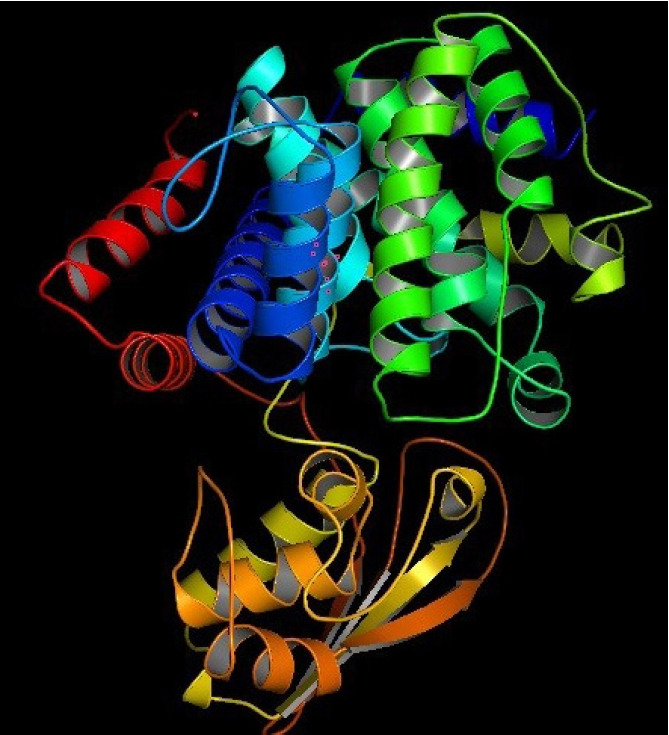
Predicted 3D structure of the hypothetical protein rendered by PyMOL.

**Fig. 5.**
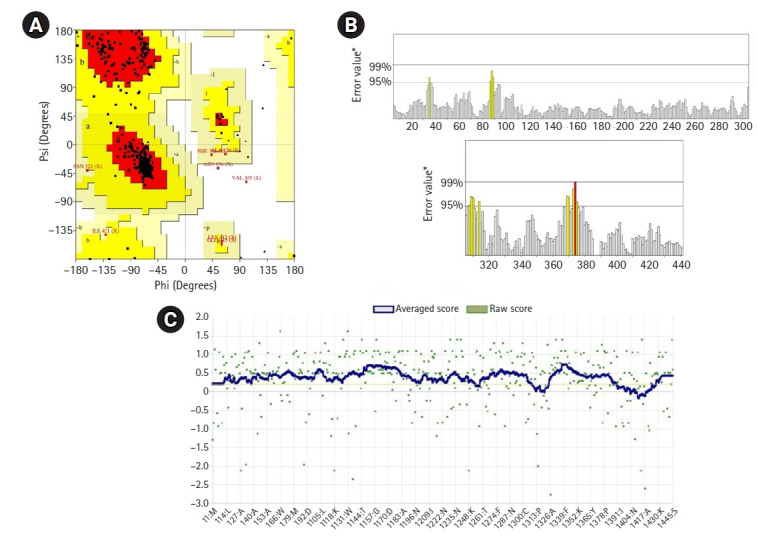
3D model of the studied hypothetical protein of *Campylobacter jejuni* validated by Ramachandran plot of PROCHECK program (A), ERRAT (B) (value overall quality factor: 96.991 from the SAVES server), and Verify3D (C).

The 3D structure of a protein is intimately connected to its functional activities. Homology modeling was used to obtain the 3D structure of the HP from HHpred. YASARA energy minimization server modified the model structure to a more stable one by reducing its energy from 11,240.6 kJ/mol to ‒219,800.0 kJ/mol. The 3D structure of the protein obtained from PyMOL (Fig. 4) was validated by PROCHECK’s Ramachandran plot analysis, Verify3D, and ERRAT. The Ramachandran plot analysis (Fig. 5A) revealed that the number of amino acids in the most favored region was 91.3% (Supplementary Table 4), which is an indicator of a valid model. An overall quality factor of 96.99 by ERRAT verified the model as good quality (Fig. 5B). Verify3D also proved the validity of the predicted model by showing that 86.52% of the residues have averaged 3D-1D score ≥ 0.2 (Fig. 5C).

### Functional annotation

The conserved domain search service tool of NCBI had identified a functional domain located in the protein sequence of the HP. The domain that was found in the HP is of MmgE/PrpD family protein (accession No. pfam03972) which is involved in propionate catabolism. Under certain conditions, the breakdown of propionate results in the creation of propionyl-CoA, which is carboxylated to D-methylmalonyl-CoA, isomerized to L-methylmalonyl-CoA, and convertes to succinyl-CoA, which is supplied to various cellular processes [32]. Many bacteria can use propionate as their only carbon source. It has a close relationship with the malonate metabolic pathway and central metabolism [33].

The result was cross-checked by InterProScan and later validated by Pfam which produced the same result. Pfam server identified MmgE/PrpD N-terminal domain at 4-440 amino acid residues with an e-value of 3.8e-105. Additionally, to identify the accuracy of the tools and databases used to specify the function of the protein, ROC curve was calculated. An average accuracy for the used pipeline was found to be 96.7% and area under the curve was 0.99 (Table 4) indicating the high reliability of *in-silico* tools and databases used in this study.

Proteins belonging to the MmgE/PrpD family protein contain 2-methylcitrate dehydratase (PrpD; 4.2.1.79). The 2-methylcitric acid cycle catalyzed by PrpD leads to propionate catabolism. PrpD catalyzes the third step of the 2-methylcitric acid cycle [34,35]. This functional protein is made up of a broad domain with an all-helical fold and a smaller domain that folds into an alpha + beta domain [36]. CAD and MmgE/PrpD family protein share a lot of similarities. In *Aspergillus terreus*, CAD is needed for the production of itaconic acid [37]. It has been previously reported that citrate/2-methylcitrate dehydratase of *Bacillus subtilis* possesses both 2-methylcitrate dehydratase and citrate dehydratase and thus it is active in the tricarboxylic acid cycle and methylcitric acid processes [38].

### PPI analysis

**Fig. 6.**
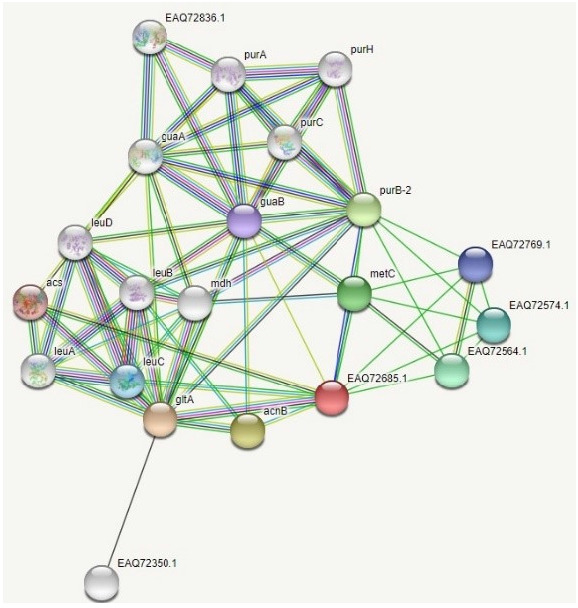
Protein-protein interaction network of the hypothetical protein from the STRING server. The colored nodes indicate the query proteins and the first shell of interactors, the white nodes indicate the second shell of interactors, the empty nodes represent proteins with an unknown three-dimensional structure, and the filled nodes represent proteins with a known or predicted three-dimensional structure.

PPI network of the HP was obtained from STRING server (Fig. 6). Functional partners with their scores predicted by the STRING search were gltA (0.991), acnB (0.961), purB-2 (0.886), metC (0.857), EAQ72564.1 (0.811), EAQ72574.1 (0.810), lecC (0.595), EAQ72769.1 (0.535), guaB (0.529), and acs (0.478) (Supplementary Table 5).

### Active site analysis

**Fig. 7.**
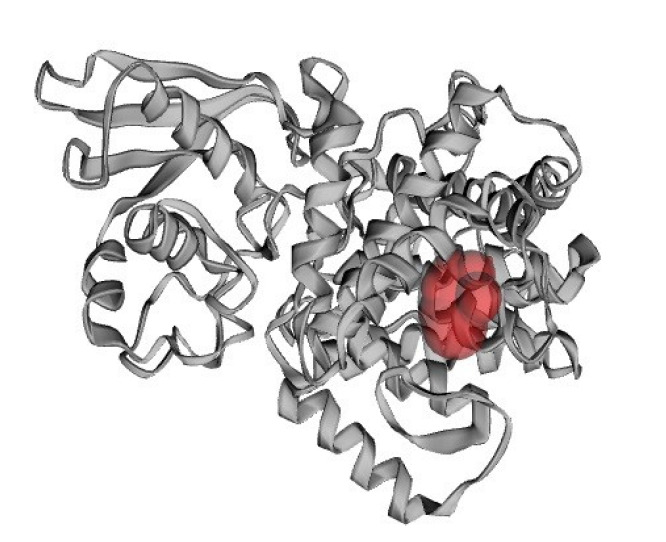
Active site (indicated as red color) of the studied hypothetical protein.

**Fig. 8.**
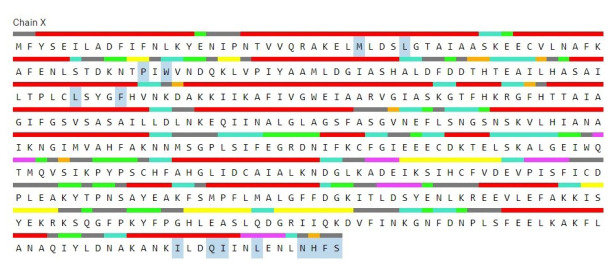
The amino acid residues in the active site of the studied protein (blue color).

Protein’s active site is the region of its surface that facilitates its binding with a specific molecular substrate which then undergoes catalysis. The CASTp server had demonstrated that 14 amino acid residues were present in the active site of the protein (Fig. 7) and the best active site was in areas with 128.249 and a volume of 79.033. The residues in the active site were shown in Fig. 8.

### Conclusion

Protein has a fundamental role in different biological processes, and all living things rely on it. The studied HP helps bacteria in propionate catabolism and influences the 2-methylcitric acid cycle. The basic knowledge on *C. jejuni* will be strengthened by these characters of the HP. However, the findings of the analyses show the validity of the bioinformatics tools and databases employed in this study, as well as the potential for extended *in-vitro* research on the HP.

## Figures and Tables

**Table 1.** List of bioinformatics tools and databases used for sequence based function annotation

Sl	Software	Function	References
A	Sequence similarity search		
1	BlastP	Used to find similar sequences in protein databases	[13]
2	MUSCLE	Used to conduct multiple sequence alignment	[14]
3	MEGA X	Used for inferring phylogenetic trees	[15]
B	Physiochemical characterization		
4	ExPASy-Protparam tool	Used for computation of various physical and chemical parameters of protein	[16]
C	Sub-cellular localization		
5	CELLO	Assign localization to both prokaryotic and eukaryotic proteins	[17]
6	PSLpred	Used to predict subcellular localization of proteins from Gram-negative bacteria	[18]
7	PSORTb	Used to predict subcellular localization of bacterial proteins	[19]
D	Secondary structure prediction		
8	SOPMA	Used to predict the secondary structure of protein	[20]
9	PSIPRED	Used for predicting PSI-blast based secondary structure to analyze protein	[21]
E	3D structure prediction and quality assessment		
10	HHpred	Used to detect protein homology by HMM-HMM comparison	[22]
11	YASARA	Utilized to increase the stability of the 3D model structure	[23]
12	PyMOL	Used for structural analysis and model figure generation	[24]
13	PROCHECK’s Ramachandran plot analysis	Used to analyze the quality and accuracy of the predicted 3D model structure	[25]
14	Verify3D	Used to assess protein’s model with 3D profiles	[26]
15	ERRAT	Used to analyze the statistics of non-bonded interactions between different atoms and verify protein structures	[27]
F	Functional annotation		
16	CD Search	Used to search for conserved structural and functional domains in a sequence	[28]
17	InterProScan	Used to search interPro for motif discovery	[29]
G	Protein-protein interaction		
18	STRING	Used for predicting protein-protein interaction	[10]
H	Active site identification		
19	CASTp	Used to find, outline, and estimate inward surface regions on protein 3D structure	[30]

**Table 2.** Similar protein obtained from non-redundant protein sequences (nr) database

Protein name	Source organism	Accession ID	Identity (%)	Score	e-value
MULTISPECIES: MmgE/PrpD family protein	*Campylobacter*	WP_002866694.1	100	910	0
MmgE/PrpD family protein	*C. jejuni*	EHD2634150.1	99.78	909	0
MmgE/PrpD family protein	*C. jejuni*	WP_057100379.1	99.78	909	0
MmgE/PrpD family protein	*C. coli*	WP_193228049.1	99.55	908	0
MULTISPECIES: MmgE/PrpD family protein	*Campylobacter*	WP_002877370.1	99.78	908	0

**Table 3.** Similar protein obtained from UniProtKB/Swiss-Prot (swissprot) database

Protein name	Source organism	Accession ID	Identity (%)	Score	e-value
Cis-aconitate decarboxylase	*Mus musculus*	P54987.2	27.06	133	5e-33
Cis-aconitate decarboxylase	*Homo sapiens*	A6NK06.1	26.91	130	6e-32
Uncharacterized protein YxeQ	*Bacillus subtilis* subsp. *subtilis* str. 168	P54956.2	23.81	128	2e-31
Cis-aconitate decarboxylase	*Aspergillus terreus*	B3IUN8.1	25.49	114	2e-26
Cis-aconitate decarboxylase	*Aspergillus terreus* NIH2624	Q0C8L3.1	25.98	113	7e-26

**Table 4.** ROC results of various tools and databases used in the present study

Tools name	Accuracy of prediction (%)	Sensitivity (%)	Specificity (%)	ROC area
BLAST	97.5	97.4	100	0.99
CD Search	95	94.9	100	0.99
InterProScan	97.5	97.4	100	0.99
Average	96.7	96.6	100	0.99

ROC, receiver operating characteristic.

## Supplementary Materials

Supplementary Table 1. List of annotated functions of 40 proteins with known function from Campylobacter jejuni using BLAST, CD Search, and InterProScan for ROC analysis

Supplementary Table 2. Hypothetical protein’s information collected from NCBI

Supplementary Table 3. Physicochemical properties of the hypothetical protein NVI_CJUN_00861

Supplementary Table 4. Ramachandran plot statistics of the hypothetical protein

Supplementary Table 5. Functional partners and their functions of the hypothetical protein predicted by the STRING server
