# Supplementary material for: In-silico characterization and structure-based functional annotation of a hypothetical protein from Campylobacter jejuni involved in propionate catabolism
Source: Genomics Inform. 2021 Dec 31;19(4):e43. doi: 10.5808/gi.21043 (PMC8752978; doi:10.5808/gi.21043)
Supplement: Supplementary Table 1. — List of annotated functions of 40 proteins with known function from Campylobacter jejuni using BLAST, CD Search, and InterProScan for ROC analysis [file gi-21043suppl1.pdf]

Supplementary Table 1. List of annotated functions of 40 proteins with known function from *Campylobacter jejuni* using BLAST, CD Search, and InterProScan for ROC analysis

Sl	Protein ID	Protein Name	BLAST[a]	CD Search[a]	InterProScan[a]
1	WP_009 881324.1	DNA polymerase III subunit beta	DNA polymerase III subunit beta 1(5)	DNA polymerase III subunit beta 1(5)	DNA polymerase III subunit beta 1(5)
2	WP_009 881354.1	Glutamate synthase large subunit	Glutamate synthase large subunit 1(5)	Glutamate synthase subunit alpha 1(5)	Glutamate synthase 1(5)
3	WP_002 855601. 1	CTP synthase	CTP synthase 1(5)	CTP synthase 1(5)	CTP synthase 1(5)
4	WP_011 812682.1	Cytochrome C biogenesis protein	Cytochrome C biogenesis protein 1(5)	Cytochrome C assembly protein 1(5)	Cytochrome C biogenesis protein 1(5)
5	WP_009 881534.1	Trans-glycosylase	Trans-glycosylase 1(5)	Trans-glycosylase 1(5)	Trans-glycosylase 1(5)
6	WP_009 881539.1	Multidrug efflux MFS transporter	Multidrug efflux MFS transporter 1(5)	Multidrug efflux MFS transporter 1(5)	Bcr/CflA family efflux MFS transporter 1(5)
7	WP_002 854281.1	Uracil-DNA glycosylase	Uracil-DNA glycosylase 1(5)	Uracil-DNA glycosylase 1(5)	Uracil-DNA Glycosylase 1(5)
8	WP_002 859393.1	Acetyl-glutamate kinase	Acetyl-glutamate kinase 1(5)	Acetyl-glutamate kinase 1(5)	Acetyl-glutamate kinase 1(5)
9	WP_002 854336.1	Molybdate ABC transporter permease	Molybdate ABC transporter permease 1(5)	Molybdate ABC transporter permease 1(5)	Molybdate ABC transporter permease 1(5)
10	WP_002 859852.1	Multidrug efflux SMR transporter	Multidrug efflux SMR transporter 1(5)	Multidrug efflux SMR transporter 1(5)	Multidrug efflux SMR transporter 1(5)
11	WP_009 882032.1	Prephenate dehydratase	Prephenate dehydratase 1(5)	Prephenate dehydratase 1(5)	Prephenate Dehydratase 1(5)
12	WP_002 858694.1	Lysine-tRNA ligase	Lysine-tRNA ligase 1(5)	Lysyl-tRNA synthetase 1(5)	tRNA synthetases 1(5)
13	WP_009 882169.1	YigZ family protein	IMPACT protein family 1(2)	IMPACT protein family 1(2)	Impact protein Family 1(2)
14	WP_002 857383.1	FAD-binding protein	FAD-binding protein 1(5)	Fumarate reductase, flavoprotein subunit 1(5)	FAD-binding Protein 1(5)
15	WP_002 782934.1	30S ribosomal protein S12	30S ribosomal protein S12 1(5)	30S ribosomal protein S12 1(5)	Uncharacterized protein 0(2)
16	WP_002 857290.1	HIT domain-containing protein	HIT domain-containing protein 1(5)	FHIT proteins, related to the HIT family 1(5)	HIT domain-containing protein 1(5)
17	WP_002 869243.1	Ferrochelatase	Ferrochelatase 1(5)	Ferrochelatase 1(5)	Ferrochelatase 1(5)
18	WP_002 854879.1	Flagellar basal body rod protein FlgB	Flagellar basal body rod protein FlgB 1(5)	Flagellar basal body rod protein FlgB 1(5)	Flagellar basal body rod protein FlgB 1(5)

19	WP_002 880964.1	Endolytic transglycosylase MltG	Endolytic transglycosylase MltG 1(5)	Cell division protein YceG, involved in septum cleavage 1(3)	Endolytic transglycosylase MltG 1(5)
20	WP_002 856958.1	ATP-binding cassette (ABC) domain-containing protein	ABC transporter permease 1(5)	ABC transporter ATP-binding protein/permease MacB 1(5)	ATP-binding cassette domain-containing protein 1(5)
21	WP_002 868904.1	TolC family protein	TolC family protein 1(5)	Outer membrane protein TolC 1(5)	TolC family Protein 1(5)
22	WP_002 869361.1	Carbamoyl-transferase HypF	Carbamoyl-transferase HypF 1(5)	Hydrogenase maturation factor HypF (carbamoyl-transferase) 1(5)	Carbamoyl-transferase HypF 1(5)
23	WP_002 869360.1	Hydrogenase formation protein HypD	Hydrogenase formation protein HypD 1(5)	Hydrogenase isoenzymes formation protein HypD 1(5)	Hydrogenase formation protein HypD 1(5)
24	WP_002 869354.1	Aspartate-tRNA ligase	Aspartate-tRNA ligase 1(5)	Aspartyl-tRNA synthetase 1(5)	Aspartate-tRNA ligase 1(5)
25	WP_002 869349.1	Major Facilitator Superfamily (MFS) transporter	MFS transporter 1(5)	MFS super family 1(5)	MFS transporter 1(5)
26	WP_002 869372.1	Molecular chaperone DnaK	Molecular chaperone DnaK 1(5)	Molecular chaperone DnaK 1(5)	Molecular chaperone DnaK 1(5)
27	WP_009 882420.1	Nucleotide exchange factor GrpE	Nucleotide exchange factor GrpE 1(5)	Heat shock protein GrpE 1(5)	Nucleotide exchange factor GrpE 1(5)
28	WP_002 857174.1	Serine O-acetyl-transferase	Serine O-acetyl-transferase 1(5)	Serine O-acetyl-transferase 1(5)	Serine O-acetyl-transferase 1(5)
29	WP_011 812734.1	ATP-dependent helicase	ATP-dependent helicase 1(5)	Superfamily I DNA or RNA helicase 1(5)	ATP-dependent helicase 1(5)
30	WP_002 867950.1	Alpha/beta hydrolase	Alpha/beta hydrolase 1(5)	Pimeloyl-ACP methyl ester carboxylesterase 1(5)	Alpha/beta hydrolase 1(5)
31	WP_002 869103.1	C-type cytochrome	None 0(2)	CytC super family 1(5)	C-type cytochrome 1(5)
32	WP_011 812744.1	DNA translocase FtsK	DNA translocase FtsK 1(5)	DNA segregation ATPase FtsK/SpoIIIE and related proteins 1(5)	DNA translocase FtsK 1(5)
33	WP_002 853404.1	GNAT family N-acetyltransferase	GNAT family N-acetyltransferase 1(5)	Acetyltransferase (GNAT) family 1(5)	GNAT family N-acetyltransferase 1(5)
34	WP_002 853451.1	RNA polymerase sigma factor RpoD	RNA polymerase sigma factor RpoD 1(5)	RNA polymerase sigma factor RpoD 1(5)	RNA polymerase Sigma factor RpoD 1(5)
35	WP_002 856550.1	Potassium transporter	Potassium transporter	Uncharacterized protein	Potassium transporter

		TrkA	TrkA<br>1(5)	0(2)	TrkA<br>1(5)
36	WP_002<br>852861.1	SsrA-binding protein SmpB	SsrA-binding protein SmpB<br>1(5)	SsrA-binding protein SmpB<br>1(5)	SsrA-binding protein SmpB<br>1(5)
37	WP_002<br>855885.1	FAD-binding protein	FAD-binding protein<br>1(5)	FAD/FMN-containing dehydrogenase<br>1(5)	FAD-binding Protein<br>1(5)
38	WP_002<br>856003.1	Riboflavin synthase	Riboflavin synthase<br>1(5)	Riboflavin synthase<br>1(5)	Riboflavin synthase<br>1(5)
39	WP_002<br>855731.1	Bacteriohemerythrin	Bacteriohemerythrin<br>1(5)	Hemerythrin<br>1(5)	Bacteriohemerythrin<br>1(5)
40	WP_002<br>869409.1	GDP-L-fucose synthase	GDP-L-fucose synthase<br>1(5)	GDP-fucose synthase<br>1(5)	GDP-L-fucose Synthase<br>1(5)

ROC, receiver operating characteristic.

[a]True positive and true negative are denoted by “1” and “0” whereas integers in () denotes the confidence level.
