# Supplementary material for: In-silico characterization and structure-based functional annotation of a hypothetical protein from Campylobacter jejuni involved in propionate catabolism
Source: Genomics Inform. 2021 Dec 31;19(4):e43. doi: 10.5808/gi.21043 (PMC8752978; doi:10.5808/gi.21043)
Supplement: Supplementary Table 2. — Hypothetical protein’s information collected from NCBI [file gi-21043suppl2.pdf]

Supplementary Table 2. Hypothetical protein's information collected from NCBI

Protein individualities	Hypothetical protein information
Locus	CAG2129885
Amino acid	446 aa
Definition	hypothetical protein NVI_CJUN_00861 [*Campylobacter jejuni*]
Accession	CAG2129885
Version	CAG2129885.1
Embl accession	CAJPVE010000003.1
Authors	Haverkamp H.A:, T.
Source strain	*Campylobacter jejuni 927*
Organism	*Campylobacter jejuni*
