# Supplementary material for: In-silico characterization and structure-based functional annotation of a hypothetical protein from Campylobacter jejuni involved in propionate catabolism
Source: Genomics Inform. 2021 Dec 31;19(4):e43. doi: 10.5808/gi.21043 (PMC8752978; doi:10.5808/gi.21043)
Supplement: Supplementary Table 3. — Physicochemical properties of the hypothetical protein NVI_CJUN_00861 [file gi-21043suppl3.pdf]

Supplementary Table 3. Physicochemical properties of the hypothetical protein NVI_CJUN_00861

Properties	Value
Molecular weight	49478.88 Da
Isoelectric point (pI)	5.93
Total number of negatively charged residues (Asp + Glu)	53
Total number of positively charged residues (Arg + Lys)	45
Molecular formula	$C_{2250}H_{3490}N_{574}O_{649}S_{16}$
Instability index	29.84
Aliphatic index	94.82
Grand average of hydropathicity (GRAVY)	–0.002
Estimated half-life	>20 h in yeast (*in-vivo*)
	>10 h in *E. coli* (*in-vivo*)
	30 h in mammalian reticulocytes (*in-vitro*)
