# Supplementary material for: In-silico characterization and structure-based functional annotation of a hypothetical protein from Campylobacter jejuni involved in propionate catabolism
Source: Genomics Inform. 2021 Dec 31;19(4):e43. doi: 10.5808/gi.21043 (PMC8752978; doi:10.5808/gi.21043)
Supplement: Supplementary Table 4. — Ramachandran plot statistics of the hypothetical protein [file gi-21043suppl4.pdf]

Supplementary Table 4. Ramachandran plot statistics of the hypothetical protein

Ramachandran plot statistics	No. (%)
Residues in the most favored regions [A, B, L]	368 (91.3)
Residues in the additional allowed regions [a, b, l, p]	27 (6.7)
Residues in the generously allowed regions [a, b, l, p]	6 (1.5)
Residues in the disallowed regions	2 (0.5)
No. of non-glycine and non-proline residues	403 (100)
No. of end-residues (excl. Gly and Pro)	2
No. of glycine residues (shown in triangles)	26
No. of proline residues	14
Total No. of residues	445
