# Supplementary material for: In-silico characterization and structure-based functional annotation of a hypothetical protein from Campylobacter jejuni involved in propionate catabolism
Source: Genomics Inform. 2021 Dec 31;19(4):e43. doi: 10.5808/gi.21043 (PMC8752978; doi:10.5808/gi.21043)
Supplement: Supplementary Table 5. — Functional partners and their functions of the hypothetical protein predicted by the STRING server [file gi-21043suppl5.pdf]

Supplementary Table 5. Functional partners and their functions of the hypothetical protein predicted by the STRING server

Functional partners	Function
gltA	Citrate synthase
acnB	Aconitate hydratase B
purB-2	Adenylosuccinate lyase
metC	Cystathionine beta-lyase
EAQ72564.1	Endoribonuclease L-PSP, putative
EAQ72574.1	Cryptic C4-dicarboxylate transporter DcuD, authentic frameshift
leuC	3-isopropylmalate dehydratase large subunit; Catalyzes the isomerization between 2-isopropylmalate and 3-isopropylmalate, via the formation of 2-isopropylmaleate
EAQ72769.1	Hypothetical protein; annotation not available
guaB	Inosine-5'-monophosphate dehydrogenase; Catalyzes the conversion of inosine 5'-phosphate (IMP) to xanthosine 5'-phosphate (XMP)
acs	Acetyl-coenzyme A synthetase; Catalyzes the conversion of acetate into acetyl-CoA (AcCoA)
